# The Role of Spontaneous Eye Blinks in Temporal Perception: An Eye Tracking Study

**DOI:** 10.3390/jemr18060076

**Published:** 2025-12-16

**Authors:** Domenica Abad-Malo, Omar Alvarado-Cando, Hakan Karsilar

**Affiliations:** 1 Psychology Brain and Innovation in Neuroscience Group, Universidad Católica de Cuenca, Cuenca 010107, Ecuador; inne@ucacue.edu.ec; 2 Bioinstrumentation and Nanomedicine Laboratory, Center for Biomedical Technology (CTB), Universidad Politécnica de Madrid, 28223 Madrid, Spain; 3 Department of Psychology, University of Groningen, Grote Kruisstraat 2/1, 9712TS Groningen, The Netherlands; h.karsilar@rug.nl

**Keywords:** time perception, spontaneous eye blinks, temporal bisection task, eye-tracking, interval timing, visual suppression

## Abstract

Our interaction with the world depends on our ability to process temporal information, which is a key component of human cognition that directly impacts decision-making, planning, and prediction of events. Visual information plays a crucial role in shaping our subjective perception of time, and even brief interruptions, such as those caused by eye blinks, can disrupt the continuity of our perception and alter how we estimate durations. The purpose of this study is to investigate the relationship between spontaneous eye blinks and time perception using a temporal bisection task. In particular, we focus on how blinks preceding stimulus presentation impact the perceived duration of that stimulus. The results of fitting a generalized linear mixed-effects model revealed that blinking can indeed influence the duration estimation. Specifically, the presence of a single blink before the stimulus presentation had a significant effect on subjective time perception; participants were more likely to categorize a duration as shorter compared to when they did not blink. In contrast, two or more blinks before stimulus presentation did not have a significant effect compared to not blinking. This study further elucidates the complex interaction between the momentary suppression of visual input and the perception of time.

## 1. Introduction

Spontaneous eye blinks occur 10 to 20 times per minute in adults, a rate that increases during childhood and stabilizes in late adolescence, representing one of the most frequently, yet unexplored, oculomotor behaviors [1,2,3,4]. Despite causing brief interruptions to visual input, blinks go mostly unnoticed due to brain mechanisms including visual suppression and perceptual continuity [5,6]. Recent research suggests that spontaneous blinks of the eyes can alter cognitive processes, including temporal perception [7,8]. Understanding how spontaneous eye blinks influence duration judgment can reveal the broader cognitive implications of oculomotor behavior and emphasizes the relationships between eye movements, brain processes, and perceptual experience [6].

During eye blinks, the perceptual system appears to ignore the gaps between blinks instead of attempting to fill them in with information [9,10]. Bristow et al. combined pupil-independent retinal stimulation with fMRI and found that blinking suppressed neural activity in the visual, parietal, and prefrontal cortex before the onset of blink [6]. This reduced brain activity was associated with the extraretinal signal, a response to the motor command of blinking, which prevents the detection of visual changes. The extraretinal signal differentiates between internal (blinks) and external (stimulus disappearance) generated visual interruptions, allowing to interpret natural and simulated blinks differently [10]. These findings highlight the remarkable ability of the brain to handle interruptions in visual input seamlessly, thus maintaining a continuous perception of the world.

In particular, spontaneous eye blinks serve as an indirect biomarker of the availability of dopamine receptors in the striatum, a brain area known to be important for temporal precision in interval timing [11,12,13]. In the context of time perception, interval timing refers to the ability to estimate durations ranging from milliseconds to seconds, which is fundamental for decision-making, motor control, and behavioral regulation [14,15,16]. A recent study by Sadibolova and colleagues confirmed that higher eye blink rates correlate with increased striatal dopamine release, which may contribute to altered temporal perception [17]. This relationship between dopamine and time perception has been well-documented in disorders characterized by dopamine dysregulation, including Parkinson’s disease, schizophrenia, autism, and attention-deficit hyperactivity disorder [18]. For instance, patients with schizophrenia have high blink rates and perceive time as passing slower, possibly due to hypervigilance [19,20,21]. Moreover, pharmacological treatments that influence dopamine receptors alter subjective time perception, as reward and timing operate using common biological pathways and are psychologically linked [22,23,24]. These findings suggest that spontaneous eye blinks are both oculomotor behavior but also a potential indicator of underlying neurochemical processes that influence cognition.

The behavioral implications of spontaneous eye blinks on temporal perception have been variable among studies, likely due to differences in blink timing relative to stimuli. Grossman and colleagues observed that blinks occurring during stimulus presentation resulted in underestimation of duration, suggesting that neural suppression occurring along blinks may lead to compression of perceived time [7]. On the other hand, Terhune and colleagues found that blinks occurring during the inter-stimulus interval between stimulus presentation and judgment screen resulted in overestimation of stimuli duration in the following trial, potentially due to fluctuations in dopamine levels that accelerate internal clock mechanisms [8]. However, Suárez-Pinilla and colleagues failed to replicate these effects using complex and dynamic videos, highlighting the role of external perceptual factors in the definition of perceived time intervals [25].

It is important to consider when interpreting these findings that individuals also engage in strategic blinking depending on task demands [26]. During stimulus presentation, people tend to suppress blinks because sustained attention is required to avoid missing critical information [27]. Hence, conclusions based on blinks during an event do not fully capture natural blinking behavior, as these blinks may be reflexive, caused by tiredness, an inherent need to pay attention, or dry eyes, rather than being truly spontaneous. In contrast, blinks that occur before stimulus onset, during preparatory or anticipatory periods, can better reflect the oculomotor patterns of baseline and their cognitive effects [28].

This study investigates how spontaneous eye blinks occurring immediately before stimulus presentation influence perceived duration. By focusing on pre-stimulus blinks, we investigate oculomotor activity under minimum task interference, giving a clearer understanding of the connection between eye blinks and time perception. We hypothesized that the presence of a pre-stimulus blink would significantly alter duration judgments compared to trials without a blink and that multiple blinks would increase this effect.

## 2. Materials and Methods

This study analyzes data from the open access repository of the Brightness and Time Perception project [29]. The analysis focuses on the constant-brightness baseline block from Experiment 2 (approximately 10 min per participant), which provided controlled conditions to examine the effects of spontaneous eye blinks on time perception.

### 2.1. Participants

A total of 30 healthy individuals (23 females, 76.7%; 7 males, 23.3%; *M*age = 20.95) participated in this study. All individuals had normal or corrected-to-normal vision. Those requiring correction were asked to use contact lenses rather than glasses to ensure data quality and minimize potential interference with the eye-tracking system. Each participant provided written informed consent before the beginning of the session. No participants were excluded from the analyses. Given the within-subject design of the study, gender composition was not expected to influence the eye blink effects under investigation.

### 2.2. Stimuli and Apparatus

The visual stimulus was a white circle displayed in the center of a 27″ LCD monitor (1920 × 1080 pixels; 60 Hz). The central area of the background was black and shifted to gray towards the sides of the screen to reduce contrast effects. The stimuli and experiment were programmed in OpenSesame 3.3 using the backend PsychoPy 2023.1.0 and the Python library PyGaze for stimulus presentation and eye-tracking, respectively [30,31,32,33]. Participants stabilized their heads on a chin rest, limiting head movements and maintaining a consistent viewing distance of 60 cm to the monitor. Responses were collected using a wired keyboard, and eye movements were recorded at 1000 Hz using the EyeLink 1000 (SR Research, Ottawa, ON, Canada) system throughout the entire experimental session. The setting was carefully controlled by using a soundproof and light-proof booth during the task, ensuring a consistent sensory environment.

### 2.3. Procedure 

**Figure 1:**
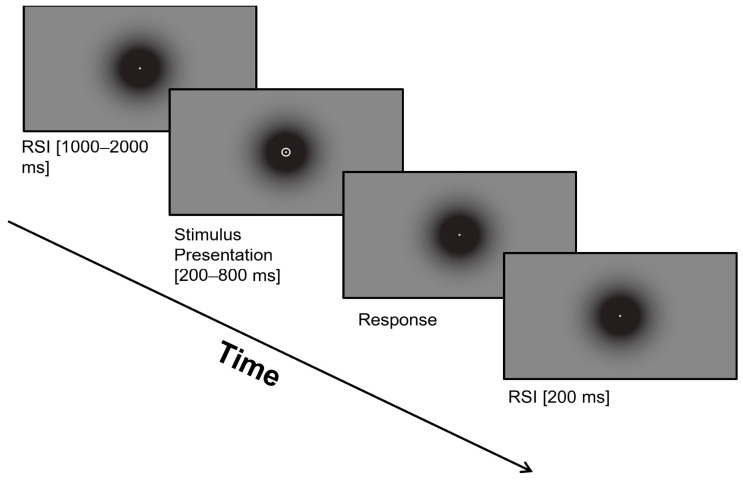
Single trial sequence in the temporal bisection task.

The experimental session began with eye tracker calibration and an explanation of the temporal bisection task that participants would perform. Participants completed a training block until they had ten consecutive correct trials; after meeting this requirement, they continued with the baseline block consisting of a total of seventy-two trials. Each trial involved three phases: fixation baseline to ensure participants focused their gaze on the dot at the center of the screen, stimulus presentation with varying probe duration, and keyboard response, categorizing stimuli as either ‘short’ or ‘long’ based on prior training, Figure 1. The inter-stimulus interval, the time between the offset and subsequent appearance of stimuli, was randomly assigned for each trial. Feedback on response accuracy was omitted to maintain natural temporal judgment without external input.

### 2.4. Temporal Bisection Task 

The task started by presenting two reference durations of the stimulus (short = 200 ms and long = 800 ms). Participants were instructed to categorize future stimuli based on these by pressing the ‘F’ and ‘K’ keys on the keyboard to indicate stimuli as ‘short’ or ‘long’, respectively. The main goal was for participants to pay attention to how long the white circle remained on the screen and determine which of the reference durations was most similar. During the training block, participants familiarized themselves with the reference durations presented randomly and with equal probability. Moving to the experimental block, participants were asked to identify both the reference durations (200 and 800 ms) and additional probe durations (320, 440, 560, 680 ms) as closer to either the short or long reference durations, using the same keyboard responses established during the training block. The probe durations of the stimuli were randomized across trials to reduce predictability and any learning effects. Each of the six probe durations was presented twelve times, resulting in a total of seventy-two trails per participant (6 durations × 12 repetitions = 72 trials). The visual stimulus, represented by a white circle, remained consistent throughout the training and testing blocks.

### 2.5. Data Processing

Of interest for this study was the number of spontaneous eye blinks that occurred immediately before stimulus presentation. The baseline for the blinking phase was set to the interval when the screen displayed a fixation cross from 1000 to 2000 ms before the stimulus presentation. The preprocessing was conducted in Python 3.11.2. Probe durations were transformed from milliseconds to seconds and were mean-centered. The dataset from Eyelink was processed using the eyelinkparser (version 0.17.3) package in Python [34]. Because the eye-tracker blink output was in arbitrary units, we categorized the data into three groups representing blink count per trial: 0 (no blink), 1 (one blink), and 2 (two or more blinks).

## 3. Results

**Figure 2:**
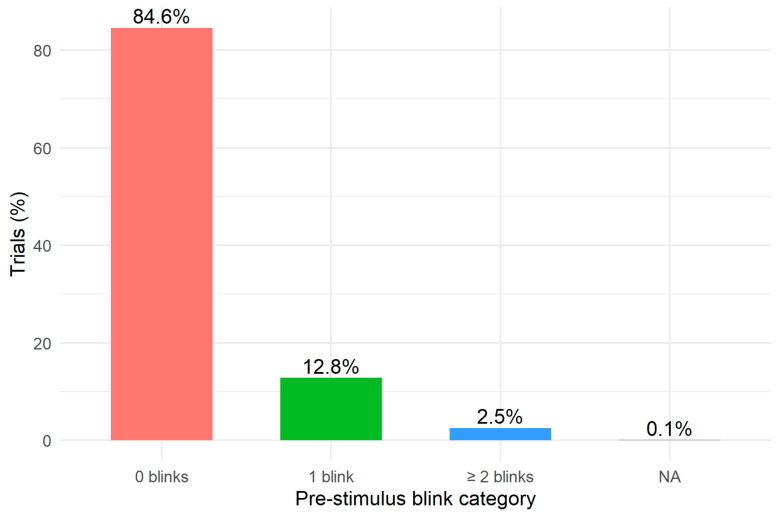
Distribution of pre-stimulus blinks.

Before running the mixed-effects model, we examined the distribution of pre-stimulus blinks. Figure 2 presents the distribution of trials across the three blink categories (0 blinks, 1 blink, ≥2 blinks). The majority of trials (84.6%) contained no pre-stimulus blinks, while 12.8% included a single blink and 2.5% included two or more blinks. This inspection confirms that blink events are relatively infrequent during the pre-stimulus interval but sufficiently represented for the inferential analysis.

Data was analyzed using a logistic generalized linear mixed-effects model (GLMER) with the participant as a random intercept. The model was fitted with the glmer function from the lme4 package in R with a binomial distribution [35,36]. The dependent variable was the probability of a ‘long’ response on each trial. The model included two fixed-effect predictors—probe duration and the number of pre-stimulus blinks—and a random intercept for participants to account for individual differences. Trials with no blink served as the reference category for blink count. This modeling method is particularly useful for behavioral studies, as it solves the multiple comparison problem and maintains trial-by-trial variance [34]. 

The fixed effects revealed statistical significance for both the intercept (β = −5.96, *SE* = 0.33, z = –18.28, *p* < 0.001) and the probe duration (β = 13.43, SE = 0.56, z = 23.83, *p* < 0.001). Regarding the effect of pre-stimulus blinks, compared to the no-blink reference category, blinking once before stimulus onset significantly decreased the probability of giving a ‘long’ response (β = −0.51, SE = 0.22, z = –2.33, *p* = 0.02). In other words, participants who blinked once before the stimulus presentation were more likely to perceive the stimulus duration as shorter compared to when they did not blink. However, the two-or-more blinks category had no significant effect on perceived duration (β = 0.21, SE = 0.47, z = 0.44, *p* = 0.66).

**Figure 3:**
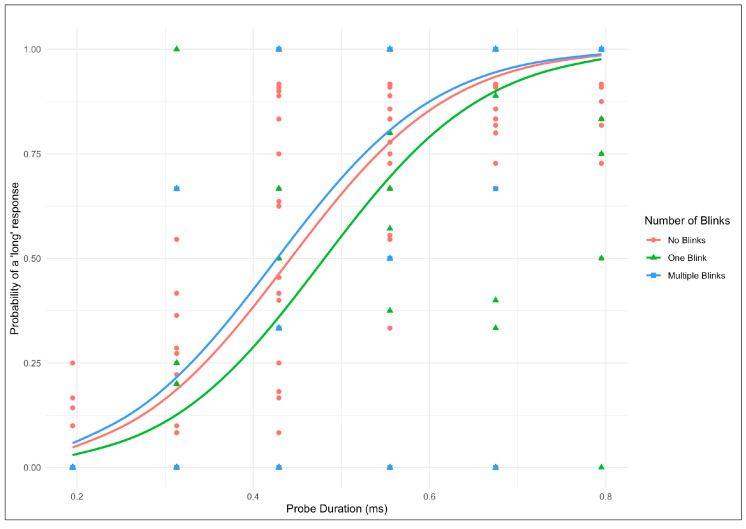
Subjective perceived time duration as a function of spontaneous eye blinks.

To complement the GLMER results, Figure 3 displays the predicted probability of giving a “long” response as a function of stimulus duration for each pre-stimulus blink category. The data points represent the observed mean responses across all participants, and the lines are the predicted probabilities derived from the GLMER model. The functions are plotted for each blink category represented by different colors, probe duration, and probability of giving a “long” response. The function reveals that a single blink before stimulus onset produces a downward shift, indicating a reduced probability of categorizing intervals as “long” compared to the no-blink baseline. This downward shift reflects the compression of perceived duration observed in the statistical analysis. In contrast, trials with two or more blinks show a pattern nearly identical to the no-blink condition, confirming the absence of additive effects.

## 4. Discussion

The purpose of this study was to investigate the influence of spontaneous eye blinks on the perceived duration of stimuli by implementing a temporal bisection task by considering both blink presence and frequency. As predicted, the results showed that blinks do indeed alter time perception. The first hypothesis was supported, as the presence or absence of blinks before the stimulus presentation had a significant effect on the judgment of duration, where participants who blinked once before the stimulus presentation were more likely to underestimate time intervals. This indicates that pre-stimulus spontaneous eye blinks influence probe duration judgment compared to instances without blinking. However, contrary to the second hypothesis, the greater number of blinks preceding stimulus onset did not have a significant effect compared to not blinking. The presence of two or more blinks before stimulus onset, as opposed to no blinks, did not affect the perceived length of that stimulus. These findings therefore argue against the suggestion that time perception and eye blink frequency have a linear relationship. Since one blink appeared to influence time judgment, this effect did not increase with more blinks.

The observed impact of a single blink on time perception is consistent with previous research showing that blinks temporarily disrupt visual continuity and have implications for altering our sense of time [7,10]. However, the absence of an effect for two or more blinks reveals an interesting yet intricate insight into timing. Specifically, it appears that more blinks do not further distort the sense of time beyond a certain threshold. One possible explanation of our findings is that participants may have cognitively compensated for multiple blinks by integrating fragmented visual information, resulting in a reduced distortion of time perception. This compensatory mechanism may have led to a perception of duration similar to trials without blinks [37]. In addition, the lack of statistical significance for the two-or-more blinks condition may be attributed to the low number of trials in this category. The short inter-stimulus interval (1000 ms to 2000 ms) made it unlikely for participants to blink multiple times before stimulus onset. Another possibility is that during high-probability periods for the stimulus, blinks were likely suppressed to avoid missing it, resulting in fewer multi-blink trials [26]. Consequently, most blinks occurred after the stimulus, creating an asymmetry in blink timing [38].

Our findings are consistent with those reported by Terhune et al. in showing that pre-stimulus blinking can influence the time perception of subsequent events [8]. However, while they reported an overestimation of the duration of the stimulus, we found an underestimation. This difference may be due to variations in the experimental design. Terhune et al. examined blinks that occurred during the inter-trial interval of the previous trial and how they influenced the duration judgments of the stimulus in the following trial [8]. In contrast, our study focused on blinks that occurred immediately before stimulus onset and their impact on the perceived duration of that same stimulus. These procedural variations (inter-stimulus intervals and stimulus being estimated) highlight the need for further research to clarify how spontaneous eye blinks influence temporal perception.

### 4.1. Limitations

While this study provides important insights into the effect of spontaneous eye blinks on time perception, it has some limitations that must be noted. First, this study assumed that all blinks recorded during the experiment were spontaneous, but it is possible that some were not truly spontaneous, possibly due to eye fatigue or dryness. Still, voluntary and simulated blinks have also been shown to alter timing, suggesting that the mechanism underlying the effect may be similar regardless of blink origin [9].

Second, the analysis focused on blinks that occur before stimulus presentation; during this period, blinks may elicit saccadic movements that could also impact time perception [38]. The current study design did not allow us to properly separate the contributions of the blink itself from the related eye movements.

Third, although blinks are believed to be an indirect measure of dopamine, the current study collected only behavioral data, so we cannot directly confirm the role of dopamine in the observed effects [11,39,40]. While the relationship between blink rate and striatal dopamine activity has been widely reported, more research employing neuroimaging or pharmacological approaches is needed to prove causality in the context of temporal perception.

Lastly, the small number of trials involving two or more blinks may have reduced statistical power to detect effects in this condition. Future studies with longer inter-stimulus intervals or longer experimental sessions may collect more multi-blink trials and provide a greater understanding of how blink frequency influences temporal judgements.

### 4.2. Future Directions

Future research should focus on key important questions to that will enable us to clarify the relationship between eye blinks and time perception. First, investigations should systematically vary blink properties such as duration, frequency, and timing in relation to stimulus presentation to determine how each factor influences timing separately. This could help clarify if the compression effect occurs due to the duration of visual suppression, the extraretinal signal, or a combination of the two.

Second, the temporal bisection task considers various aspects of timing, including the assessment of stimulus duration, the storage and maintenance of duration information in memory, and the comparison of durations [41]. Future studies combining this behavioral paradigm with neuroimaging techniques such as functional magnetic resonance imaging (fMRI) or electroencephalography (EEG) could reveal relevant details about neural mechanisms underlying these processes and clarify the involvement of dopamine pathways. These studies could directly identify if the reported behavioral effects are mediated by striatal activity.

Third, future studies should examine the conditions under which blinks alter time estimation and reproduction, as these tasks may use different mechanisms. Time perception does not rely on a single dedicated sensory organ; rather, it emerges from integrating sensory inputs, such as auditory and visual information [42,43,44]. Looking into how blinks influence duration judgments across multiple modalities (auditory, tactile) could reveal if the effect is limited to visual timing or has a broader impact on temporal processing.

## 5. Conclusions

This study demonstrates that spontaneous eye blinks occurring immediately before stimulus presentation alter temporal perception. We provide empirical evidence that brief losses of visual input—even a single spontaneous blink, lasting milliseconds—can shorten the perceived duration of a subsequent visual stimulus. In contrast, multiple blinks in succession did not produce additional distortions in time perception. These findings illustrate the complex interplay of sensory and cognitive processes in timing, suggesting that temporal perception is not solely determined by the physical properties of stimuli but is also influenced by the ongoing state of the oculomotor system.

These findings may have theoretical implications, suggesting that models of subjective time perception should integrate both neurobiological and behavioral mechanisms. On a practical level, a better understanding of blinking behavior might be beneficial in domains like human-computer interaction and building technological systems requiring precise timing. Additionally, these findings could inform clinical approaches for populations with dysregulated dopamine pathways and impaired timing abilities. Continuing research into the mechanisms underlying these effects will help us understand how brief visual suppression and neurochemical processes interact to alter our perception of time.

## Data Availability

The original data presented in the study are openly available in Brightness and Time Perception at https://osf.io/9gkbw/overview (accessed on 1 December 2025).
